# Comparative study between airway pressure release ventilation (APRV) and biphasic positive airway pressure (BIPAP) ventilation for patients with acute respiratory distress syndrome (ARDS)

**DOI:** 10.62838/jccm-2026-0057

**Published:** 2026-10-30

**Authors:** Ahmed Fayez Abdelraouf, Galal Abouelsoud Saleh, Manal Mohamed Kamal, Rana Ayman Mohamed, Niven Gerges Fahmy

**Affiliations:** Department of Anesthesia, Intensive care and Pain Management, Faculty of Medicine, Ain Shams University, Cairo, Egypt

**Keywords:** airway pressure release ventilation, biphasic positive airway pressure, acute respiratory distress syndrome

## Abstract

Severe hypoxemia, decreased respiratory system compliance, and extensive inflammatory infiltrates are the hallmarks of acute respiratory distress syndrome, that commonly requires mechanical ventilation. The best ventilator strategies for ARDS are still being researched, despite advancements in ventilatory control. Pressure-controlled ventilation modes, including airway pressure release ventilation and biphasic positive airway pressure ventilation, are frequently used in clinical practice and can affect oxygenation, respiratory mechanics, and mechanical ventilation duration through a variety of physiological mechanisms.

**Aim:**

To compare APRV and BiPAP modes regarding oxygenation, respiratory mechanics and clinical outcomes in intubated ventilated ARDS patients.

**Methods:**

Fifty adult patients with ARDS were recruited in this study after admission to the ICU and initiation of mechanical ventilation. They were randomly assigned to two groups: Group A (APRV) and Group B (BiPAP).

**Results:**

The two groups were comparable in terms of age, sex, and hemodynamics throughout study time points. Oxygenation and partial pressure of carbon dioxide, baseline respiratory mechanics (plateau pressure, mean airway pressure and compliance) were comparable between the two groups. Thereafter, mean airway pressure became higher in the APRV group throughout further study time points; however, the differences were statistically significant only on days 1 and 3. Patients in the APRV group required significantly higher doses of fentanyl and midazolam compared with the BiPAP group on day 1 and 3 only. No significant differences were observed between the two groups in ventilation duration, ventilator-free days by day 28, ICU length of stay, clinical complication occurrence or mortality.

**Conclusion:**

In ARDS patients, no significant difference observed between APRV mode and BiPAP mode regarding oxygenation, respiratory mechanics, mechanical ventilation duration and occurrence of clinical complications.

## Introduction

Sepsis, pneumonia, aspiration, and trauma are major causes of ARDS, a very harmful disease of the lung that results in low blood oxygen levels. Although mechanical ventilation has markedly evolved, ARDS continues to be associated with a high mortality rate (40%). Management of ARDS commonly includes lung protective ventilation using low tidal volume ventilation (LTVV), optimal positive end-expiratory pressure, permissive hypercapnia, recruitment maneuvers, and prone positioning [1,2].

Although LTVV is considered the standard approach for ARDS patients nowadays, it carries the risk of decreasing lung inflation which may lead to hypoxemia and atelectasis [3].

Lung recruitment techniques may lessen ventilation associated lung injury and lower the significant risk of mortality for ARDS patients, whereas alveolar stress and strain are increased by atelectasis during low tidal volume ventilation. On the other hand, high airway pressure application might damage the pulmonary epithelium (barotrauma) and compromise the cardiovascular function. As a result, there is debate about the use of lung recruitment techniques [4].

BiPAP is a technique that reduces breathing effort and encourages inspiration [3]. This partial support mode uses time-cycled, pressure-controlled ventilation with two levels of continuous positive airway pressure (CPAP) and permits spontaneous respiratory effort, which may take place throughout the entire respiratory cycle. In order to adjust the inspiratory effort, BiPAP may change the frequency of regulated breaths [5].

The inspiratory to expiratory ratio is not inverse in conventional biphasic breathing. This ratio is often used to differentiate APRV from biphasic ventilation. The inspiratory positive airway pressure (IPAP), expiratory positive airway pressure (EPAP), inspiratory time, respiratory rate, and fraction of inspired oxygen (FiO_2_) are among the ventilator parameters that are set in BiPAP mode [6].

According to the open lung concept, APRV is a continuous positive airway pressure with a short intermittent release phase. It also fully aligns with the ARDS therapy principles. Despite this, APRV has historically only been used as a salvage technique for ARDS patients with inadequate oxygenation [7].

APRV mode, which uses two pressure levels with inverted ratio ventilation, permits spontaneous breathing. While increased baseline pressure enhances oxygenation, release of airway pressure during APRV mode promotes expiration [8].

Both BiPAP and APRV are examples of intermittent mandatory ventilation with pressure targeting that alternates between higher and lower pressure levels. Unlike biphasic positive airway pressure, APRV provides constant high pressure (Phigh) at a preset of a longer inspiratory time (Thigh) with sporadic releases to a lower pressure level (Plow) at a preset of a shorter expiratory time (Tlow). The goal of this reduced expiratory duration with APRV is to produce an intrinsic positive end-expiratory pressure (PEEP), which should improve alveolar recruitment and lessen lung cyclic opening and collapse [9].

APRV has been advocated as a way to enhance patient prognosis in light of a better awareness of the pathogenesis of ARDS in recent years [7].

## Methods

### Study population

Between February 2024 and March 2025, fifty patients were assigned randomly in 1:1 ratio into two groups: group A (was managed by APRV) and group B (was managed by BiPAP).

Adult patients who met all diagnostic criteria for ARDS based on the Berlin definition were included in the research. Prior to recruitment, eligible patients required ventilatory support and endotracheal intubation for fewer than 48 hours. They also had a PaO_2_/FiO_2_ ratio of 200 or below.

Patients under the age of 18 or above the age of 65 or with underlying illnesses that might affect respiratory or clinical results like rheumatic or ischemic heart disease, suspected or confirmed intracranial hypertension, cancer or immunosuppressive therapy use, pregnancy, neuromuscular disorders associated with prolonged intubation, chronic pulmonary diseases (e.g., COPD, suppurative pulmonary infections, diffuse alveolar hemorrhage, or evidence of barotrauma), and cardiac disease were excluded.

### Randomization and allocation concealment

Eligible patients who met the inclusion criteria were randomly assigned to either the APRV group or the BiPAP group using consecutively numbered, opaque, sealed envelopes. The allocation sequence was generated in advance using a computer-generated random block design. Each envelope contained the treatment assignment and was opened only after patient enrollment.

Due to the nature of the ventilatory strategies, blinding of the ICU team was not feasible; however, the outcomes consisted mainly of objective physiological and ventilatory measurements, which are less susceptible to observer bias.

### Ethical Considerations

Participants’ relatives in the ICU were freely given fully informed consent to participate. Written informed consents were used in this prospective investigation. Data security and participant confidentiality were ensured. Anytime along the study procedure, participants should be allowed to request removal of their data. The ethics committee of Ain Shams University’s medical faculty approved this work.

### Study Procedures

APRV parameters were:

- High airway pressure (Phigh) was set according to the most recently measured airway plateau pressure (Pplat), and was limited to a maximum of 30 cmH2O, while low airway pressure (Plow) was maintained at (0 -5 cmH2O).
- THigh was configured to occupy 90 % of the total respiratory cycle (between 4 and 6 seconds) and modified based on patient’s oxygenation and clinical response.
- Release time (Tlow) was initially adjusted to approximately 1–1.5 times the expiratory time constant, and then titrated to end expiration when expiratory flow at 50–75% of the peak expiratory flow rate (PEFR).
- The release frequency was maintained within the range of 10–14 cycles per min.
- BiPAP parameters were:
- Based on the inspiratory time constant, inspiratory time (Ti) was modified.
- PEEP was titrated to obtain adequate oxygenation, targeting arterial PaO_2_ 60–80 mmHg (or SpO2 > 90 %).
- FiO_2_ was titrated to maintain SpO2 ≥ 90%.
  » The objective of mechanical ventilation in both groups was to keep plateau airway pressure (Pplat) below 30 cmH2O.
  » To reach the desired degree of patient comfort, analgesia and sedation were administered in both groups in a comparable manner. A Richmond Agitation–Sedation Scale score (RASS) between −2 and 0 was used to reach the sedation goal. Every four hours (or more often when clinically indicated) RASS scores were measured and recorded. Analgesic and sedative drug doses were then modified to maintain the desired range. To maintain spontaneous breathing in the APRV group, ventilator settings together with sedative and analgesic dosage titration were adjusted by physicians.
  » Patients were withdrawn from the study if they developed any life-threatening complications during the study period, including severe hypoxemia unresponsive to the assigned ventilation strategy (PaO_2_/FiO_2_< 100 despite at least 12 hours of invasive mechanical ventilation), refractory hypoxemia (PaO_2_< 55 mmHg with FiO_2_ of 1.0), refractory (barotrauma, severe respiratory acidosis, or shock).
  » Vasopressors were given in line with the institutional ICU protocol and titrated to maintain an appropriate mean arterial pressure.

### Outcomes

- Baseline and daily oxygenation (PaO_2_/FiO_2_) and respiratory mechanics: “plateau pressure, mean airway pressure and compliance “were recorded.
- Baseline and daily measurement of hemodynamics were recorded.
- Baseline and daily recording of arterial blood of carbon dioxide PaCO2 were recorded.
- Ventilator-free days by day 28.
- ICU stay duration.
- Sedation requirements needed for tolerating mechanical ventilation were recorded.
- Clinical complication (pneumothorax).
- Mortality during ICU stay for each group was recorded.
  » Baseline refers to measurements obtained immediately after initiation of the assigned ventilatory mode.

### Statistical methods

The acquired data was processed, tabulated, and analyzed statistically by IBM SPSS statistics (Statistical Package for Social Sciences) software. Quantitative data was normalized using the Shapiro-Wilk test, reported as mean±SD (standard deviation), minimum and maximum range, then compared using an independent t-test. Qualitative data was reported in terms of number and percentage, and the Chi square test was applied to compare them. A p-value of less than 0.050 was identified as significant, otherwise was non-significant.

## Results

The study flow is illustrated in Figure 1 (CONSORT diagram).

**Figure 1:**
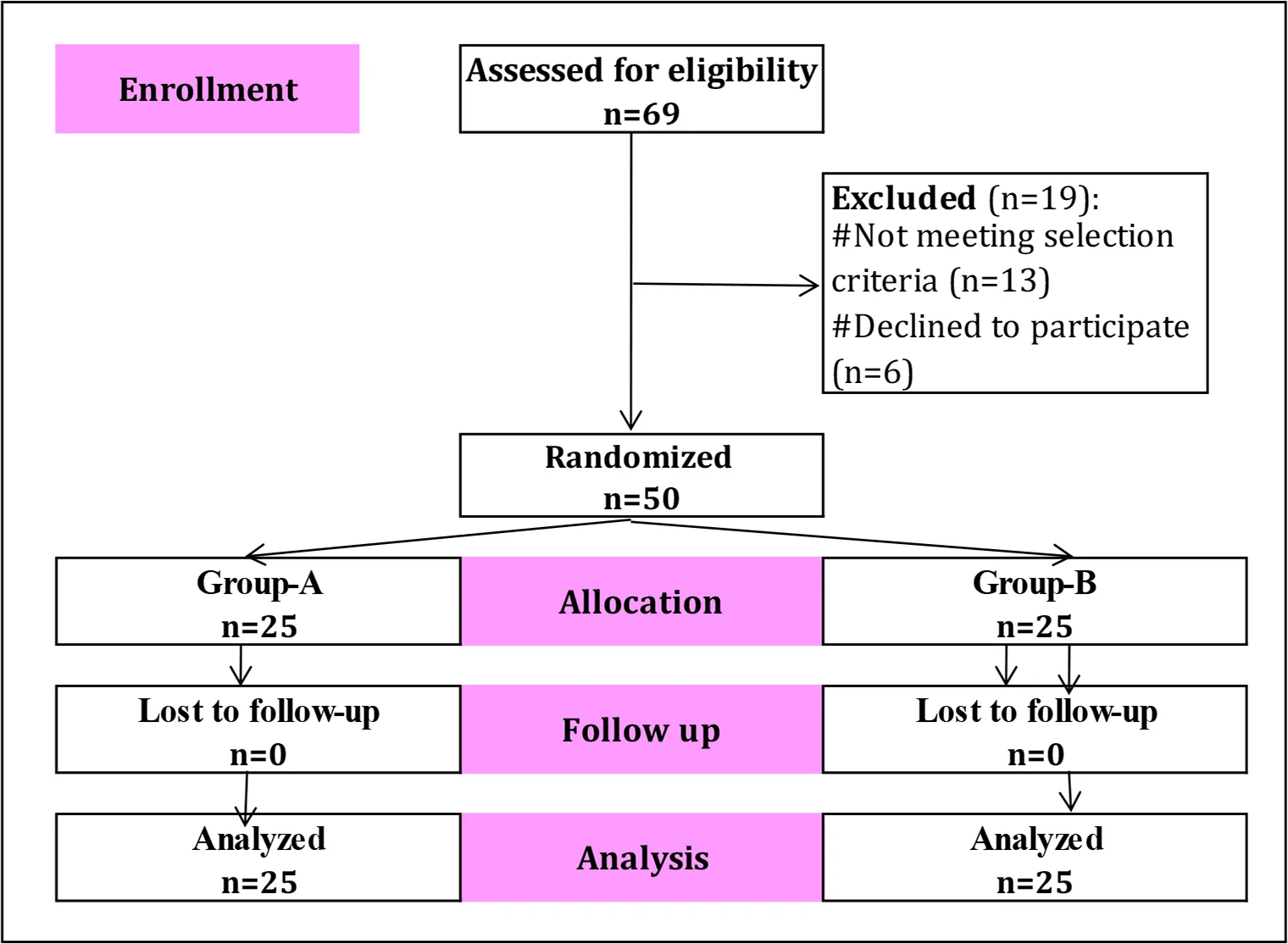
Study flow diagram (CONSORT)

**Table (1):** Demographic data of the studied groups

**Variables**		**APRV group (n=25)**	**BiPAP group (n=25)**	**p-value**
Age (years)		46.5±7.9	47.2±8.6	^^^0.772
Sex	Male	17 (68.0%)	16 (64.0%)	^#^0.765
	Female	8 (32.0%)	9 (36.0%)	
BMI (kg/m^2^)		29.4±2.4	28.9±2.2	^^^0.445

Data are presented as Mean±SD or (n) number of patients (%). BMI= Body Mass Index.

# Chi square test.

^ Independent t-test.

**Table (2):** Heart rate and mean arterial pressure between the study groups

**Time**	**APRV group (n=25)**	**BiPAP group (n=25)**	**^p-value**	**Relative effect**	
				**Mean±SE**	**95% CI**
Heart rate (beat/minute)					
Baseline	111.4±6.1	109.1±4.6	0.134	−2.3±1.5	−5.4–0.7
Day-1	108.0±7.4	105.6±6.4	0.219	−2.4±2.0	−6.4–1.5
Day-3	93.2±7.1	93.5±6.1	0.888	−0.3±1.9	−4.0–3.5
Day-5	79.3±9.1	80.3±6.6	0.680	−0.9±2.3	−5.5–3.6
Day-7	73.5±6.1	75.3±4.9	0.266	−1.8±1.6	−4.9–1.4
Mean arterial pressure (mmHg)					
Baseline	81.6±6.7	80.8±5.3	0.578	−1.0±1.7	−4.4–2.5
Day-1	83.3±7.2	86.5±7.2	0.121	−3.2±2.0	−7.3–0.9
Day-3	86.1±7.6	89.0±7.1	0.172	−2.9±2.1	−7.1–1.3
Day-5	89.0±9.3	91.4±7.7	0.334	−2.4±2.4	−7.2–2.5
Day-7	93.3±6.5	95.5±5.5	0.195	−2.2±1.7	−5.7–1.2

Data are presented as Mean±SD.

^ Independent t-test. SE= Standard error. CI= Confidence interval. Relative effect= Effect in APRV group versus BiPAP group.

**Table (3):** Oxygenation (PaO_2_/FiO_2_) and arterial blood of carbon dioxide PaCO2 between the study groups

**Time**	**APRV group (n=25)**	**BiPAP group (n=25)**	**^p-value**	**Relative effect**	
				**Mean±SE**	**95% CI**
PaO_2_/FiO_2_					
Baseline	129.2±13.7	131.9±11.3	0.439	−2.8±3.5	−9.9–4.4
Day-1	147.5±28.2	137.5±16.9	0.134	10.0±6.6	−3.2–23.3
Day-3	161.7±22.9	160.5±20.1	0.840	1.2±6.1	−11.0–13.5
Day-5	205.1±21.3	197.9±21.8	0.240	7.2±6.1	−5.0–19.5
Day-7	222.7±13.5	220.1±11.8	0.473	2.6±3.6	−4.6–9.8
PaCO_2_ (mmHg)					
Baseline	53.0±1.2	52.7±1.2	0.418	0.3±0.3	−0.4–1.0
Day-1	51.6±1.3	52.2±1.8	0.183	−0.6±0.4	−1.5–0.3
Day-3	48.9±1.5	49.6±1.5	0.114	−0.7±0.4	−1.5–0.2
Day-5	46.3±1.8	47.0±2.0	0.212	−0.7±0.5	−1.8–0.4
Day-7	42.5±1.4	43.2±1.5	0.102	−0.7±0.4	−1.5–0.1

Data are presented as Mean±SD.

^ Independent t-test. SE= Standard error. CI= Confidence interval. Relative effect= Effect in APRV group versus BiPAP group.

**Table (4):** Respiratory mechanics: plateau pressure, mean airway pressure and compliance between the study groups

**Time**	**APRV group (n=25)**	**BiPAP group (n=25)**	**^p-value**	**Relative effect**	
				**Mean±SE**	**95% CI**
Plateau pressure (cmH_2_O)					
Baseline	26.9±1.8	27.3±2.0	0.438	−0.4±0.5	−1.4–0.6
Day-1	24.7±2.3	24.0±2.2	0.255	0.7±0.6	−0.5–2.0
Day-3	24.2±1.8	23.6±2.2	0.264	0.6±0.6	−0.5–1.8
Day-5	23.5±2.1	22.3±2.5	0.072	1.2±0.7	−0.1–2.5
Day-7	22.9±2.2	22.1±2.4	0.225	0.8±0.7	−0.5–2.1
Mean airway pressure (cmH_2_O)					
Baseline	28.0±1.1	27.7±1.2	0.347	0.3±0.3	−0.4–1.0
Day-1	27.1±1.8	24.6±2.2	<0.001*	2.6±0.6	1.4–3.7
Day-3	26.0±1.8	23.7±2.3	<0.001*	2.3±0.6	1.1–3.5
Day-5	21.0±2.5	19.7±2.0	0.062	1.2±0.6	−0.1–2.5
Day-7	19.7±1.5	19.0±1.3	0.082	0.7±0.4	−0.1–1.5
Compliance (ml/cmH_2_O)					
Baseline	34.1±0.8	34.0±1.0	0.763	0.1±0.3	−0.5–0.6
Day-1	34.7±1.1	34.4±0.8	0.184	0.4±0.3	−0.2–0.9
Day-3	35.3±1.1	35.0±1.2	0.330	0.3±0.3	−0.3–1.0
Day-5	36.9±1.2	37.1±1.7	0.569	−0.2±0.4	−1.1–0.6
Day-7	37.8±0.8	37.6±1.2	0.492	0.2±0.3	−0.4–0.8

Data are presented as Mean±SD.

^ Independent t-test. SE= Standard error. CI= Confidence interval. Relative effect= Effect in APRV group related to that in BiPAP group.

**Table (5):** Fentanyl infusion rate (mcg/kg/h) between the study groups

**Time**		**APRV group (n=25)**	**BiPAP group (n=25)**	**^p-value**	**Relative effect** **Mean±SE** **95% CI**
Baseline	Mean±SD Range	1.20±0.07 1.10–1.30	0.80±0.10 0.60–1.00	0.001	0.40±0.02 0.35–0.45
Day-1	Mean±SD Range	1.01±0.12 0.80–1.20	0.61±0.11 0.40–0.80	0.001	0.40±0.03 0.34–0.46
Day-3	Mean±SD Range	0.86±0.11 0.70–1.10	0.52±0.08 0.40–0.70	0.001	0.34±0.03 0.29–0.39
Day-5	Mean±SD Range	0.50±0.14 0.30–0.70	0.44±0.09 0.30–0.60	0.055	0.06±0.03 0.00–0.13
Day-7	Mean±SD Range	0.46±0.09 0.20–0.50	0.42±0.09 0.30–0.60	0.084	0.04±0.02 −0.01–0.09

^ Independent t-test. SE= Standard error. CI= Confidence interval. Relative effect= Effect in APRV group relative to that in BiPAP group.

**Table (6):** Midazolam infusion rate (mg/kg/h) between the study groups

**Time**		**APRV group (n=25)**	**BiPAP group (n=25)**	**^p-value**	**Relative effect** **Mean±SE** **95% CI**
Baseline	Mean±SD	0.24±0.02	0.19±0.02	0.001	0.06±0.01
	Range	0.22–0.29	0.16–0.23		0.05–0.07
Day-1	Mean±SD	0.21±0.02	0.18±0.02	0.001	0.03±0.00
	Range	0.17–0.23	0.14–0.22		0.02–0.04
Day-3	Mean±SD	0.19±0.02	0.16±0.01	0.001	0.03±0.00
	Range	0.14–0.22	0.14–0.19		0.02–0.04
Day-5	Mean±SD	0.14±0.03	0.12±0.03	0.058	0.02±0.01
	Range	0.09–0.21	0.06–0.17		0.00–0.04
Day-7	Mean±SD	0.12±0.05	0.09±0.05	0.054	0.03±0.01
	Range	0.03–0.24	0.03–0.21		0.00–0.06

^ Independent t-test. SE= Standard error. CI= Confidence interval. Relative effect= Effect in APRV group related to that in BiPAP group.

**Table (7):** Clinical outcomes between the study groups

**Variables**		**APRV group (n=25)**	**BiPAP group (n=25)**	**^p-value**	**Relative effect** **Mean±SE / Relative risk** **95% CI**
Ventilation duration (days)	Mean±SD	8.4±1.9	8.2±1.8	0.650	0.2±0.5
	Range	5.0–12.0	5.0–12.0		−0.8–1.3
Ventilator-free days by day 28	Mean±SD	19.6±1.9	19.8±1.8	0.650	−0.2±0.5
	Range	16.0–23.0	16.0–23.0		−1.3–0.8
Length of ICU stay (days)	Mean±SD	10.4±1.8	10.1±1.9	0.543	0.3±0.5
	Range	7.0–14.0	7.0–15.0		−0.7–1.4
Pneumothorax (n, %)		3 (12.0%)	2 (8.0%)	§0.999	1.50 0.27–8.22
Mortality (n, %)		2 (8.0%)	2 (8.0%)	§0.999	1.00 0.15–6.55

§ Fisher’s Exact test.

^ Independent t-test. SE= Standard error. CI= Confidence interval. Relative effect= Effect in APRV group related to that in BiPAP group.

- On days 1 and 3, sedation requirements by (fentanyl and midazolam) were significantly greater in APRV group than BiPAP group, suggesting a greater need for sedation during the early phase of APRV support. By days 5 and 7, sedation requirements decreased in both groups with no significant difference observed, indicating a convergence in patient comfort and ventilator synchrony over time.

## Discussion

This study yielded several important results. Study outcomes were monitoring patient’s oxygenation, respiratory mechanics, hemodynamics and PaCO2, duration of intensive care unit stay, number of ventilator free days by day 28, sedation requirements and occurrence of clinical complications or mortality.

Regarding respiratory mechanics, prior meta-analyses have demonstrated improved respiratory system compliance and higher mean airway pressure (MAWP) with APRV compared with conventional LTVV [1]. The higher MAWP observed with APRV reflects sustained alveolar recruitment during prolonged Thigh, which theoretically reduces cyclic trauma.

In the present study, although MAWP was significantly elevated in the APRV group in the first 3 days, respiratory system compliance didn’t differ significantly between APRV and BiPAP.

This study results are in line with those of Stoll et al., who found that patients associated with COVID-19 related ARDS had increased MAWP in APRV as opposed to BiPAP. This study’s lack of a comparable compliance benefit raises the possibility that better recruitment and oxygenation might take place without appreciable compliance modifications, especially in mild ARDs [9].

In the current study, oxygenation parameters didn’t differ significantly between APRV and BiPAP groups. This suggests that both modes when optimally applied, can achieve comparable oxygenation targets in ARDS patients.

This finding contrasts with meta-analysis by Othman et al., which reported significantly higher PaO_2_/FiO_2_ ratios on day 3 in patients receiving APRV compared to conventional ventilation. Differences in comparator modes (BiPAP versus volume- controlled ventilation), timing of assessment and patient populations may explain this discrepancy [14].

The need for sedation varies across investigations. In a cross-sectional research involving 40 ARDS patients, Salem SS discovered that the BIPAP group needed fewer days of sedation than the SIMV PC (synchronized intermittent mandatory ventilation with pressure control) group during the course of the trial [11]. Additionally, Compared to APRV, BiPAP was linked in our research to less early sedation requirements. This might be a sign of patient discomfort brought on by greater sustained airway pressures during APRV, especially in the early stages of adjustment.

However, Zhou et al., reported lower sedation requirements with APRV during the first week of ventilation, likely attributable to preserved spontaneous breathing and improved patient-ventilator synchrony once patients were adequately adapted to the mode [10].

The current study demonstrated no statistically marked difference in PaCO2 levels between patients managed with APRV and those receiving BiPAP. This finding suggests that when appropriately set, both ventilatory modes can achieve comparable alveolar ventilation despite their fundamentally different mechanics.

Theoretical concerns regarding hypercapnia during APRV stem from the intentionally brief release phase (Tlow), which limits tidal ventilation and may predispose to CO2 retention. However, preservation of spontaneous respiration during the prolonged high-pressure phase may enhance alveolar ventilation and decrease hypercapnia. This could explain why PaCO2 levels in our cohort remained comparable to those observed with BiPAP.

Previous studies have reported conflicting results. Ibarra Estrada et al., described a higher incidence of transient severe hypercapnia in patients receiving APRV, particularly in COVID-associated ARDS, where lung compliance and dead space are often markedly impaired [12]. In contrast, Zou et al., reported significantly lower PaCO2 values in patients ventilated with APRV compared to LTVV [15].

Taken together, these findings suggest that hypercapnia during APRV isn’t an inherent limitation in the mode itself but rather depends on disease severity, spontaneous breathing contribution, and meticulous adjustment of release time.

The APRV and BiPAP groups did not significantly vary in the incidence of pneumothorax in the current investigation. The idea that persistent pressures would be less harmful than recurrent alveolar opening and collapse is supported by the lack of enhanced barotrauma, even if APRV is associated to greater MAWP.

In this study, 3 patients out of 25 in the APRV group suffered from pneumothorax representing (12%), while in BiPAP group; 8% of the study patients suffered from pneumothorax.

This finding supports the safety of APRV when lung protective principles are followed and is aligned with the findings described by Hirshberg et al. acute respiratory failure patients [13].

This study has several limitations. The relatively small sample size may have reduced the power to detect significant differences in some clinical outcomes. Being conducted at a single center, the generalizability of the findings to other institutions may be limited. Blinding was not feasible due to the visible nature of ventilatory modes. Future multicenter trials with larger sample sizes are warranted to confirm these findings.

## Conclusion

In ARDS patients, no significant difference observed between APRV mode and BiPAP mode regarding oxygenation, respiratory mechanics, mechanical ventilation duration and occurrence of clinical complications.

## References

[1] Zhong X, Wu Q, Yang H (2020). Airway pressure release ventilation versus low tidal volume ventilation for patients with acute respiratory distress syndrome/acute lung injury: A meta-analysis of randomized clinical trials. Annals of Translational Medicine..

[2] Al-Khalisy H, Nieman GF, Kollisch-Singule M (2024). Time-Controlled Adaptive Ventilation (TCAV): A personalized strategy for lung protection. Respiratory Research..

[3] Khayat S, Ali M, Almasoudi L (2022). The impact of BiLevel Positive Airway Pressure (BiPAP) application timing on emergency room length of stay in patients with pulmonary edema: A single-center, retrospective cohort study. Cureus..

[4] Goligher EC, Hodgson CL, Adhikari NK (2017). Lung recruitment maneuvers for adult patients with acute respiratory distress syndrome: A systematic review and meta-analysis. Annals of the American Thoracic Society..

[5] Saddy F, Moraes L, Santos CL (2013). Biphasic positive airway pressure minimizes biological impact on lung tissue in mild acute lung injury independent of etiology. Critical Care..

[6] Gallagher JJ (2018). Alternative modes of mechanical ventilation. AACN Advanced Critical Care..

[7] Cheng J, Ma A, Dong M (2022). Does airway pressure release ventilation offer new hope for treating acute respiratory distress syndrome ?. Journal of Integrative Medicine..

[8] Abo bakr H, Taha Ab, Ahmed A (2019). Comparative study between airway pressure release ventilation and synchronized intermittent mandatory ventilation in treatment of acute respiratory distress syndrome. The egyptian journal of hospital medicine..

[9] Stoll SE, Leupold T, Drinhaus H (2025). Comparison of airway pressure release ventilation (APRV) versus biphasic positive airway pressure (BIPAP) ventilation in COVID-19-associated ARDS using transpulmonary pressure monitoring. BMC Anesthesiology.

[10] Zhou Y, Jin X, Lv Y (2017). Early application of airway pressure release ventilation may reduce the duration of mechanical ventilation in acute respiratory distress syndrome. Intensive Care Medicine..

[11] Salem SS, Hussein K, Badawy MS (2023). Biphasic positive airway pressure in the management of acute respiratory distress syndrome: A comparative study. Egyptian Journal of Bronchology.

[12] Ibarra-Estrada MA, García-Salas Y, Mireles-Cabodevila E (2022). Use of airway pressure release ventilation in patients with acute respiratory failure due to COVID-19: Results of a single-center randomized controlled trial. Critical Care Medicine..

[13] Hirshberg EL, Lanspa MJ, Peterson J (2018). Randomized feasibility trial of a low tidal volume-airway pressure release ventilation protocol compared with traditional airway pressure release ventilation and volume control ventilation protocols. Critical Care Medicine..

[14] Othman F, Alsagami N, Alharbi R (2012). The efficacy of airway pressure release ventilation in acute respiratory distress syndrome adult patients: A meta-analysis of clinical trials. Ann Thorac Med..

[15] Zou X, Zhang H, Wu Y (2025). Physiologic Comparison of Airway Pressure Release Ventilation and Low Tidal Volume Ventilation in ARDS. A Randomized Controlled Trial. Chest..

